# CRISPR/Cas9 in Planta Hairy Root Transformation: A Powerful Platform for Functional Analysis of Root Traits in Soybean

**DOI:** 10.3390/plants11081044

**Published:** 2022-04-12

**Authors:** Mohsen Niazian, François Belzile, Davoud Torkamaneh

**Affiliations:** 1 Département de Phytologie, Université Laval, Québec City, QC G1V 0A6, Canada; mohsen.niazian.1@ulaval.ca (M.N.); francois.belzile@fsaa.ulaval.ca (F.B.); 2 Institut de Biologie Intégrative et des Systèmes (IBIS), Université Laval, Québec City, QC G1V 0A6, Canada; 3 Field and Horticultural Crops Research Department, Kurdistan Agricultural and Natural Resources Research and Education Center, Agricultural Research, Education and Extension Organization (AREEO), Sanandaj 6616936311, Iran

**Keywords:** functional analysis, nodulation, reverse genetics, targeted mutation, transformation

## Abstract

Sequence and expression data obtained by next-generation sequencing (NGS)-based forward genetics methods often allow the identification of candidate causal genes. To provide true experimental evidence of a gene’s function, reverse genetics techniques are highly valuable. Site-directed mutagenesis through transfer DNA (T-DNA) delivery is an efficient reverse screen method in plant functional analysis. Precise modification of targeted crop genome sequences is possible through the stable and/or transient delivery of clustered regularly interspaced short palindromic repeat (CRISPR)/CRISPR-associated protein (CRISPR/Cas) reagents. Currently, CRISPR/Cas9 is the most powerful reverse genetics approach for fast and precise functional analysis of candidate genes/mutations of interest. Rapid and large-scale analyses of CRISPR/Cas-induced mutagenesis is achievable through *Agrobacterium rhizogenes*-mediated hairy root transformation. The combination of *A. rhizogenes* hairy root-CRISPR/Cas provides an extraordinary platform for rapid, precise, easy, and cost-effective “in root” functional analysis of genes of interest in legume plants, including soybean. Both hairy root transformation and CRISPR/Cas9 techniques have their own complexities and considerations. Here, we discuss recent advancements in soybean hairy root transformation and CRISPR/Cas9 techniques. We highlight the critical factors required to enhance mutation induction and hairy root transformation, including the new generation of reporter genes, methods of *Agrobacterium* infection, accurate gRNA design strategies, Cas9 variants, gene regulatory elements of gRNAs and Cas9 nuclease cassettes and their configuration in the final binary vector to study genes involved in root-related traits in soybean.

## 1. Introduction

In recent years, forward genetic methods have enabled the identification of candidate genes and enzymes involved in a trait or biosynthetic pathway of interest, owing to the fast development of large-scale sequencing techniques, thus leading to a much-increased availability of genome and transcriptome data [1]. These powerful forward genetic techniques have allowed the identification of many quantitative trait loci (QTL) as well as candidate genes and even candidate causal mutations controlling a specific trait [2]; however, the dissection of the exact function of these genetic components remains challenging. In parallel, reverse genetic approaches, such as recombinant DNA technology and mutagenesis, have been applied for functional annotation of genetic components, causal mutations, and candidate genes identified by forward genetic techniques [3]. Reverse genetic methods play a complementary, but necessary role in next-generation sequencing (NGS)-based forward genetic approaches to precisely describe the function of candidate genetic components and subsequently improve agronomic traits [4].

In the past few years, recombinant DNA technology has shown a great advantage over random mutagenesis, where obtaining a desired mutant requires generating and screening large mutant populations. Moreover, reduced viability is another problem associated with induced mutagenesis [2]. On the contrary, site-directed mutagenesis through recombinant DNA technology and the insertion of T-DNA (vector-based mutagenesis) represents a sophisticated method for functional gene analysis and the development of new traits [5]. These approaches require fast and efficient transformation techniques to generate transgenic plants/organs. To date, different transformation methods, including *Agrobacterium*-mediated, imbibition, biolistic, osmotic, liposome, microinjection, pollen tube pathway, shoot apex, infiltration, and silicon carbide-mediated transformation [6,7,8], have been developed to transfer exogenous genetic components to a plant’s genome [9]. Currently, *Agrobacterium*-mediated gene transfer is the most effective and prevalent method [9,10], as it can precisely integrate a DNA sequence into an active section of the host genome with a low copy number and consistent gene expression over generations [11].

Overall, transformation can be conducted in stable or transient manners. Stable transformation refers to the stable integration of exogenous DNA into the host genome, whereas transient transformation is the short-term (transient) expression of gene constructs, resident bacterial plasmid, carrying exogenous DNA in a plant cell. For stable transformation, whole-plant in vitro regeneration is required, which is a time-consuming and costly process. Direct organogenesis, indirect organogenesis, or somatic embryogenesis are the main routes of in vitro regeneration in legumes [12]. Currently, direct organogenesis using cotyledonary nodes or other meristematic explants (e.g., apical/axillary meristems) is the most prevalent in vitro regeneration method for *Agrobacterium*-mediated transformation of legume species [12]. However, some legumes, such as soybean (*Glycine max* L.), are recalcitrant to stable transformation because of limited in vitro regeneration capacity and low DNA transfer rates [1].

In contrast, transient transformation, with no need for complete plant regeneration, provides an efficient alternative for rapid and large-scale functional analysis in soybean plants [13]. This approach has no effect on the stability of the host genome and is independent of the positional effects of the T-DNA integration sites [14]. Therefore, chimeric and composite plants (containing wild-type shoots with transgenic roots) can be generated in an efficient manner, and transgenic roots, with a “hairy root” phenotype, can be analyzed to assess the function of genes of interest, especially genes involved in legume-rhizobium symbiosis and root–microbe interactions [15,16]. Most interestingly, *Agrobacterium rhizogenes*-mediated transformed roots can be used to regenerate whole transformed plants. In general, composite plants can be obtained through in vitro and or in planta (ex vitro) inoculation techniques [17]. Ex vitro hairy root induction is completely independent from laborious in vitro techniques and has great advantages for large-scale gene validation studies. This is especially the case for the functional characterization of genes suspected of controlling root traits. These include genes involved in plant metabolic engineering, plant-pathogen interactions, symbiotic nitrogen fixation (SNF) and nodulation, biotic and abiotic stress tolerance, mycorrhization, phytoremediation, root-shoot interaction, nutrient uptake, and hormone transport [18].

Problems, such as the random insertion of the DNA construct into loci in chromosomes and copy number variability, are the limitations associated with conventional vector-based mutagenesis in both stable and transient transformation methods [19]. Induction of DNA double-strand breaks (DSBs) at specific genome locations using targeted genome editing technologies (site-directed nucleases) has provided plant scientists with a great opportunity to create desired mutations in a more precise way [5]. Chimeric enzymes, such as zinc-finger nucleases (ZFNs) [20] and transcription activator-like effector nucleases (TALENs) [21], harbor both a domain specifying the target sequence and an endonuclease domain to affect a DSB at the desired site in the genome. In contrast, the CRISPR/Cas system relies on two distinct molecular entities: a guide RNA (gRNA) to specify the target site and a Cas endonuclease to cut the DNA [22,23]. Although these entities provide efficient targeted genome editing methods in plant systems, they are not without challenges. The high cost and difficulty in developing amino acid motifs that bind to the desired target sequence with high affinity and specificity are the main bottlenecks encountered with ZFNs and TALENs [24]. Unlike ZFNs and TALENs, the CRISPR/Cas9 approach relies on base pairing between nucleic acids to ensure the targeting of a specific site in the genome. This approach has been rapidly improved for targeted genome editing in plants, and there is a day-to-day development of this technique in different plant species. The CRISPR/Cas9 method has been widely used to manipulate different genes involved in fatty acid and protein synthesis, seed weight, seed size, plant height, plant vigor (e.g., node and stem development), flowering time, biotic and abiotic stress resistance, and symbiosis in soybean [25,26].

Like conventional T-DNA transformation, stable and/or transient delivery of CRISPR/Cas9 components, embedded in a binary vector, is required for the induction of targeted mutations in plants. Both *A. tumefaciens*- and *A. rhizogenes*-mediated transformation methods are applicable for the delivery of CRISPR/Cas9 reagents to plant cells. A combination of transient *A. rhizogenes*-mediated ex vitro hairy root induction and the CRISPR/Cas9 technique is a revolutionary method for fast and precise functional validation of root-related candidate genes/mutations [27]. Recent improvements and challenges of transient hairy root transformation and the CRISPR/Cas9 system will be discussed in the following sections.

## 2. Hairy Root Transformation

As a “natural genetic engineer”, *A. rhizogenes* has the ability to transfer the T-DNA region of its Ri plasmid (root-inducing plasmid) into the plant’s genome [28]. The injection of this plasmid into the host cells and stimulation of the production and development of highly branched hairy roots are the consequence of the expression of a group of *rol*/oncogenes (*rolA*, *rolB*, *rolC,* and *rolD*) in the bacterial Ri plasmid [29,30]. These *rol* genes are involved in hairy root syndrome induction through different biochemical functions, such as phytohormone homeostasis, reactive oxygen species (ROS) homeostasis, and ornithine cyclodeaminase. These properties make *rol* genes a powerful tool in plant biotechnology and functional biology. Another important application of *rol* genes is the in vitro production of valuable plant bioactive compounds, such as secondary metabolites [9]. Co-transformation of the Ri plasmid, containing *rol* genes in its T-DNA region, along with an expression (disarmed binary) vector containing the gene of interest in the T-DNA region, made *A. rhizogenes* a fast, simple, and highly efficient system to analyze the function of genes involved in root traits in the transformed hairy roots [15,31] in both model and non-model plant species [18]. As every transgenic root represents an independent transformation event, a high transformation efficiency (percentage of induced hairy roots harboring the transferred gene(s) of interest) is achievable by *A. rhizogenes*-mediated hairy root transformation in a relatively short period of time [32].

*A. rhizogenes*-mediated hairy root induction has been conducted using both in vitro and in planta inoculation methods in soybean [32,33]. Inoculation of cotyledon explants in an *A. rhizogenes* solution is the most commonly used in vitro method for hairy root transformation in soybean. Parameters such as *A. rhizogenes* strain, *Agrobacterium* cell density, plant genotype, inoculation and co-cultivation duration, root induction media and its composition, as well as the type and concentration of *Agrobacterium*-killing antibiotics are important to reaching high hairy root transformation efficiency [33]. To date, different *A. rhizogenes* strains (i.e., A4, LBA9402, R1000, K599 (NCPPB2659), ARqual, and A4RS) were used for hairy root induction in different plant species [27]. Treatments such as vacuum infiltration and sonication have also been used in the in vitro inoculation method to increase the transformation efficiency [9]. *Agrobacterium* injection is also applicable under in vitro conditions [1].

An efficient in vitro *A. rhizogenes*-mediated hairy root transformation technique in soybean was presented by Chen et al. [33]. This method consists of the inoculation of 5-day-old cotyledons, wounded in the attachment points of cotyledons and hypocotyls, in an *Agrobacterium* suspension (strain K599) for 30 min (with shaking at 50 r min^−1^ at 28 °C) followed by five days of co-cultivation on filter paper. The transformation efficiency of this method for delivery of a reporter gene was 30–60% [33]. Different modifications of this technique (e.g., different optical densities of *Agrobacterium*, different combinations of co-culture medium) have been reported in recent soybean hairy root transformation experiments [34,35,36].

In planta *A. rhizogenes* inoculation is the most often reported method of soybean transformation using a hairy root system. In vitro inoculation and its required aseptic conditions have been eliminated from in planta techniques, which make them faster, less labor-intensive, and more cost-effective. In this method, the *Agrobacterium* strain used and the age of young seedlings at the time of *Agrobacterium* introduction are influential factors [32]. Rockwool inoculation, *Agrobacterium* dipping, and *Agrobacterium* injection are three applicable in planta techniques; however, *Agrobacterium* injection is the most prevalent method. Hypocotyl and/or the cotyledonary node are the most used tissues for in planta hairy root induction in soybean [37]. Most in planta *A. rhizogenes* studies in soybean are based on the protocol reported by Kereszt et al. [32]. This protocol is based on infection of cotyledonary nodes and/or hypocotyls of five-day-old seedlings with *Agrobacterium* paste and/or an *Agrobacterium* suspension. Some modifications, such as infection of the central part of the hypocotyl of younger seedlings (2–4 d old), have been reported for this protocol [38,39].

Chimeric root systems formed by *Agrobacterium rhizogenes* transformation are composed of both transformed and non-transformed roots, as a different percentage of transformation can occur, and reaching 100% transformation efficiency is impossible [40]. Therefore, identifying which of the induced hairy roots are transgenic is a critical step. In general, the collection of hairy roots, the identification of transformed roots, and their re-introduction into pots are the main steps of hairy root transformation [40]. Currently, it is possible to use different reporter genes for the visual detection of transgenic roots. The α-glucuronidase (*GUS*) reporter gene is the most used reporter gene in hairy root transformation studies [41]. However, it is a destructive method, as it requires staining. In most cases, gene functional analysis studies using *A. rhizogenes*-mediated hairy root induction require a non-destructive in vivo identification system. Fluorescent proteins provide an interesting option in this regard. The green fluorescent protein (*GFP*) is a prevalent reporter gene used for the visual detection of transgenic tissues (e.g., [42,43]). However, it is not suitable for lignified or flavone-containing tissues as their autofluorescence emission overlaps with GFP, and this background interference makes it difficult for the in vivo detection of GFP. For this reason, *GFP* is not a perfect in vivo reporter for flavone-rich roots like soybean roots. Alternatively, the red fluorescent protein (DsRed2) has high solubility in plant tissues, minimizes background interference (significantly different emission spectrum from that of the autofluorescence of plant root tissue), and is resistant to photo-bleaching, which makes it a better reporter gene [44,45]. In addition, because of its small size (671 bp), manipulation of the *DsRed2* gene in vector constructs is relatively easy [41]. Despite its advantages, the DsRed2 system requires a fluorescence microscope for detection [41]. Luckily, there are some new reporter genes that are directly visible to the naked eye. *RUBY* is a new reporter gene that converts tyrosine to vividly red betalain. Transformed roots can be identified visually without any chemical treatments or special equipment [46]. *RUBY* is a valuable reporter gene for non-invasively monitoring hairy roots, as roots are not photosynthetic tissues and there is no concern about the interference of the red pigment produced with the green pigments of photosynthetic organs [46]. *eYGFPuv* is another reporter gene that has recently been successfully used for identifying transgenic roots under UV light [47].

## 3. CRISPR/Cas9 System for Functional Analysis in Soybean

Transgene-induced overexpression or suppression (RNAi-based knock-down) of a target gene are two important reverse genetic methods to understand a gene’s role. Both overexpression and knock-down have been achieved through hairy root transformation in soybean [48,49,50,51,52,53]. There are some pros and cons with both systems. The unpredictable nature of T-DNA insertion sites, and the associated position effects that can impact the level of expression of the introduced transgene, is an important disadvantage of vector-based overexpression studies [54]. Targeted suppression of desired genes in a homology-dependent manner using RNAi is a good way to assess the function of identified candidate genes [55]. RNAi is a quick, easy, and cost-effective sequence-specific method for validating gene function. There are, however, potential pitfalls in this approach, such as limited sequence specificity of siRNAs (off-target activity), transitive silencing, inefficacy and instability, and difficulty in the validation of RNAi knock-down (as the wild-type transgene would be silenced and validation by complementation with a wild-type gene is impossible) [56]. Targeted genome editing methods can provide more stable and predictable mutant lines to investigate gene function [56]. Among these, ZFNs and TALENs have seen few applications in legumes because of the complexity of designing DNA-binding modules, their inefficiency in genome targeting, and their prevalent off-target activities [57]. Currently, the CRISPR/cas9 system is the most popular method of targeted genome editing for functional analysis of genes in legumes [54], especially in soybean with a paleopolyploid genome background [58].

**Figure 1:**
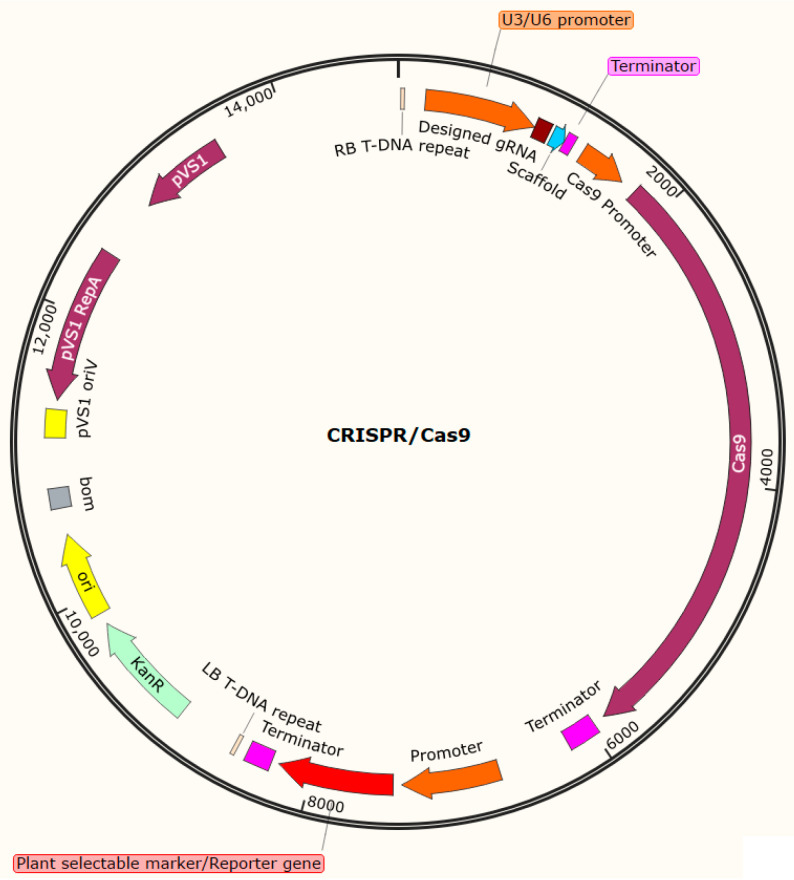
The schematic representation of a binary vector harboring gRNA, Cas9, and selectable marker/reporter gene cassettes in its T-DNA region for targeted genome editing of plants.

Simply put, the whole CRISPR/Cas9 genome editing procedure is based on an RNA-guided Cas9 DNA cleavage mechanism [3,59,60,61]. Therefore, a CRISPR/Cas9 construct consists of gene coding for the Cas9 nuclease and an expression cassette for at least one guide RNA (gRNA) embedded in the T-DNA region of a binary vector (Figure 1). The subsequent repair of Cas9-induced DSBs by the imprecise non-homologous end-joining (NHEJ) repair process will lead to insertions/deletions (indels), many of which will result in a non-functional or dysfunctional protein. Recent reviews have extensively described CRISPR/Cas9-based genome editing [59,62,63].

An efficient editing procedure requires optimization of the Cas9 gene and gRNA expression. In general, there are four factors that should be considered in designing a CRISPR/Cas9 genome editing construct, (i) gRNA design, (ii) selection of a suitable Cas9 protein, (iii) gene regulatory elements (GREs) of the Cas9 nuclease and gRNA cassette, and (iv) configuration of the gRNA and Cas9 cassettes inside the binary vector. Some important aspects for optimizing CRISPR/Cas9 reagents to achieve a greater genome editing efficiency and reducing experimental failure are discussed in the following sections.

### 3.1. gRNA and Its Components

A CRISPR RNA (crRNA) and a transactivating CRISPR RNA (tracrRNA) are the two components of a single guide RNA (sgRNA) in the natural CRISPR/Cas9 system. In the engineered version of the CRISPR/Cas9 system, the crRNA spacer sequence will be replaced with the designed target sequence at the time of gRNA cassette preparation. The important part of the spacer sequence is the seed sequence that binds to the target DNA, following recognition of the protospacer-adjacent motif (PAM), and therefore it is required for precise target recognition and binding. Depending on the Cas variant (Section 3.5), the length of the seed sequence varies from 5–12 nt upstream of the PAM. Mismatches between the seed sequence of a gRNA and the targeted region constitute an important risk for failure in CRISPR activity [64]. The second part of a gRNA—the scaffold sequence—is responsible for generating the required secondary structure to bind to the target sequence and is considered the constant part of a gRNA.

### 3.2. Critical Criteria in Designing gRNA

#### 3.2.1. Proper Selection of the Target Site(s) within the Locus of Interest

Depending on the desired outcome of editing, different regions within a targeted gene/locus can be selected for mutation induction. In knock-out experiments via a premature termination codon (PTC) strategy, targeting earlier exons is more effective than exons very close to an ATG or intron–exon junction, as PTC-induced loss of function is not common in these regions [64,65].

#### 3.2.2. Determination and Prediction of Off-Target Activity

Success in the induction of desired mutations and preventing off-target editing are two key outcomes of a successful genome editing experiment. These experiments require that great care be taken in designing the gRNA. The cleavage efficiency of candidate gRNAs can be determined in a faster manner through an in vitro screening method rather than through in vivo expression [66]. Predicting off-target activities is another approach to finding the most efficient gRNAs among different candidates. Some of the web-based software for designing gRNAs present information regarding the genome-wide off-target activities of the candidate gRNA [64]. Machine learning algorithms can also predict the mutation induction efficiency of a designed gRNA sequence using repair data obtained from previous studies [67,68]. Although care in designing an optimal site-specific gRNA is the best way to reduce off-target activity, there are some wet-lab procedures that can be used to reduce the frequency of off-target events; these include: (i) reducing gRNA-Cas9 concentration, (ii) using double-nicking mediated by a Cas9 nickase mutant (nCas9), and (iii) using truncated gRNAs (tru-gRNAs) [69].

#### 3.2.3. Nucleotide Features of the Designed gRNA and its Associated Secondary Structure

A GC content of 30–80% and no mismatch to the targeted sequence, especially in the seed region targeting the non-transcribed strand, have been reported as key features of an effective gRNA [64]. Another aspect that should be considered is avoiding multiple “Ts” in the construct [64]. Repeat and anti-repeat (RAR) stem-loop, stem-loop 1, stem-loop 2, and stem-loop 3 are four required stem-loop structures that should be present in an effective gRNA. The formation of a functional Cas9-gRNA-DNA complex does not necessarily depend on the stem-loop 1 structure. However, stem-loops RAR, 2, and 3 are required and important in plant genome editing experiments [70]. In silico analysis of gRNA secondary structure through web-based software, such as RNA-fold (http://rna.tbi.univie.ac.at/cgi-bin/RNAWebSuite/RNAfold.cgi (accessed on 1 February 2022), is an appropriate procedure to predict the efficiency of a designed gRNA [71]. In addition to the predicted secondary structure, information about the free energy (ΔG) of the self-folding potential of the designed gRNA is available in the RNA-fold WebServer. A designed gRNA with self-folding free energy within the range of 0 to 2.0 kcal/mol can lead to the highest cleavage efficiency of Cas9 [71].

### 3.3. Number of Designed gRNAs

Designing at least two independent gRNAs for each target gene is a solution to minimize the risk of experiment failure in a CRISPR/Cas9 experiment [72]. Designing multiple gRNAs is especially important in species such as soybean, where nearly 75% of the genes are present in multiple copies, so one gRNA is not enough for simultaneous mutation induction in paralogs [57]. Multiple designed gRNAs should be individually inserted between the promotor and gRNA scaffold of a gRNA cassette using several rounds of standard restriction-ligation cloning, which is a tedious and time-consuming procedure [70]. In the following section, we highlight different strategies for assembling multiple gRNAs.

### 3.4. Assembly of Multiple gRNAs and gRNA Processing Strategies

In multiplex gene editing studies, multiple gRNA cassettes designed to target different genes or different regions of a gene at once should be assembled as a single unit and inserted in the destination vector. The first strategy is the insertion of the designed and synthesized double-stranded oligonucleotides in individual gRNA vectors, then isolation of each gRNA cassette (promotor + guide sequence + gRNA scaffold + terminator) from gRNA vectors and their assembly in an intermediate/destination construct using Golden Gate cloning. In Golden Gate assembly, gRNA cassettes should be designed in a way to produce compatible overhanging ends after digestion with type IIS restriction enzymes. After ligation of adjacent units, the resulting gRNA module can then be mobilized into the binary vector of interest using different cloning methods. Zheng et al. [73] provided a Golden Gate-based modular system that can assemble 2, 4, and 6 gRNA units for targeted mutagenesis of soybean [73]. Gibson assembly is another technique for the assembly of various gRNA cassettes and is independent from end-compatibility issues and PCR cleanup steps [74]. The GoldenBraid cloning system has also been used to assemble multiple gRNAs and insert them into destination binary vectors [75]. Modification of existing CRISPR vectors for substitution of other Cas9 proteins, fluorescence proteins, and required resistance genes can be easily performed by using the GoldenBraid cloning system [70,76]. For gene knock-out experiments using gene editing technology, the GoldenBraid technique is well suited as it can carry multiple gRNA constructs leading to an increased possibility of mutagenesis [77]. This technique can also be used for complete disruption of the genes of interest by introducing large deletions [64].

Cloning multiple gRNA cassettes as independent units can present disadvantages such as frequent recombination events and plasmid instability in *E. coli* and *Agrobacterium* because of repeated promotor sequences (promoter crosstalk effects) [78]. Therefore, the configuration of multiple gRNAs and their simultaneous expression as a single transcript can be more beneficial. In this case, a single polycistronic transcript will be cleaved into individual gRNAs post-transcriptionally using different RNA processing strategies [79]. The CRISPR-associated RNA endoribonuclease Csy4 (from *Pseudomonas aeruginosa*), the tRNA-processing endogenous enzymes, and self-processing ribozymes can be used for post-transcriptional cleavage of a polycistronic transcript [78]. Luo et al. [80] used a polycistronic approach (with Csy4 used to achieve cleavage) and reported a 45.3% mutation induction efficiency when targeting *GW2* paralogs in soybean. A polycistronic tRNA-gRNA (PTG) vector is another processing strategy for multiplexing gRNAs [79]. Similarly, cleavage of the tRNAs by endogenous self-processing ribozymes, such as RNaseP and RNaseZ, can be used to release multiple gRNAs from a longer transcript. Depending on the preferred RNA processing strategy, multiplex Csy4-gRNAs, tRNAs-gRNAs, and/or ribozyme-gRNAs can join in a single reaction using Golden Gate modular cloning or Gibson assembly [79,81]. Multiplexing PTG system has been successfully applied to target different genes in soybean [82,83].

### 3.5. Cas9 Nucleases

The Cas protein is responsible for DNA cleavage in a targeted region of the plant genome. Therefore, a high-fidelity Cas enzyme can increase the specificity and efficiency of a targeted genome editing procedure. *Streptococcus pyogenes* Cas9 (SpCas9) is the most robust and widely used Cas enzyme [59]. The presence of a canonical PAM sequence (NGG) in the targeted genomic loci is, however, a prerequisite for cleavage by SpCas9. Therefore, there is a limitation in the number of genomic loci that can be targeted by this protein [84]. Fortunately, different Cas9 variants, with distinct PAM specificities, can be used to expand this range [85]. In a study, three variants of Cas9 (xCas9, SpCas9-NG, and XNG-Cas9) were assessed in soybean [46]. Authors reported that xCas9 was successful with the NGG and KGA PAMs, SpCas9-NG recognized NGD (NGG, NGA, and NGT), RGC (AGC or GGC), GAA, and GAT PAM sites, whereas XNG-Cas9 cleaved only regions with NGG, GAA, and AGY PAM sequences [46]. These variants of Cas9 can be used to induce mutations in targets devoid of NGG PAMs.

After choosing the most appropriate Cas9 variant, some additional actions are required to increase its efficiency, such as codon optimization, adding nuclear localization signals (NLSs), and the insertion of introns. Grützner et al. [86] compared the efficiency of different Cas9 nucleases in *Arabidopsis*: a human codon-optimized Cas9 with a C-terminal NLS, a maize codon-optimized Cas9 with a C-terminal NLS and with/out an additional N-terminal NLS, and a zCas9 with 13 *Arabidopsis* introns in its sequence that contains a C-terminal NLS with/out an additional N-terminal NLS. They showed that constructs with introns work better than those without introns and two NLSs seem better than one. In the same work, it was found that a Cas9 with introns also proved superior in *Nicotiana benthamiana* and *Catharanthus roseus*.

### 3.6. Chimeric Deactivated Cas9 Proteins and Their Applications

The Cas9 enzyme can cleave DNA fragments because of its RuvC and HNH nuclease domains. Point mutations in the nuclease domains of SpCas9 can inactivate its RuvC and HNH domains and create a modified Cas9 protein [87]. The D10A mutation (in the RuvC domain) and the H840A mutation (in the HNH domain) both create a nickase enzyme (nCas9). Together, these two mutations result in a nuclease-dead Cas9 (dCas9), which is unable to cleave target DNA while retaining its ability to bind to target DNA with the help of a gRNA [87]. Fusion of nCas9 or dCas9 peptides to other proteins or protein domains can enable other DNA modifications at targeted loci. Such chimeric proteins can be used for specific purposes such as base editing, transcriptional repression, epigenetic modification, and in vivo labeling (reviewed in [68]).

Among these applications, base editing can be used for the generation of specific allelic mutants and single nucleotide polymorphisms (SNPs) to evaluate their functional impact [88]. Multiplex base alterations can be used to create multiple substitutions, thus facilitating the directed evolution of plant genes [78]. In a base editing procedure, gRNAs should be designed such that the targeted base is located between positions 4 and 8 in the gRNA sequences (editing window), counting the end distal to the PAM as position 1 [89]. Two CBEs have been conducted in soybean, and their results showed that the editing window of nCas9-APOBEC1 is located between positions 5–7 in the gRNA sequence, counting the end distal to the PAM as position 1 [90,91].

### 3.7. Gene Regulatory Elements (GREs) of gRNAs and Cas9 Nuclease Cassettes

Promotors and terminators used in gRNA and Cas9 cassettes are the other critical parameters for achieving an efficient genome edition. Finding the appropriate promoters is crucial as off-target activities can increase markedly when concentrations of the Cas9-gRNA complex are excessive [64]. Two types of RNA polymerase III promoters, including U6/U3, are the most commonly used promoters to express gRNAs in plants [69]. Finding the appropriate variant(s) of U6/U3 promoters is an important step in optimizing gRNA expression as there are various versions of these promoters [64]. Endogenous U6/U3 promoters can lead to better editing outcomes than foreign promoters. In soybean, editing efficiency was significantly increased when the GmU6 promoter (14.7–20.2%) was used instead of the AtU6 promoter (3.2–9.7%) [69]. Similar results were reported when comparing the GmU6-16g-1 promoter (43.4–48.1%) to the AtU6-26 promoter (11.7–18.1%) via soybean hairy-root transformation [92].

Different types of promoters can be used: (i) constitutive (from viruses or plants), (ii) tissue-specific, (iii) inducible, and (iv) developmentally regulated promoters. Constitutive promoters of viral origin (CaMV35S and NOS) derived from plant housekeeping genes (UBIQUITIN or ACTIN) are the most commonly used. Broadly, higher efficiency of plant-derived promoters has been reported [64].

Terminators are other GREs that can affect the stability of Cas9 and gRNA transcripts. RNA Pol II readthrough can interfere with RNA Pol III-mediated transcription of gRNAs can happen when the Cas9 cassette has a weak terminator and both the Cas9 and gRNA expression cassettes are in the same orientation [64]. This can be minimized by using a strong terminator in the Cas9 transcription unit or using an opposite orientation of the gRNA and Cas9 expression cassettes (head-to-head) in the binary vector [65]. A comparative analysis of different terminators revealed that the rbcS-E9 terminator (from *Pisum sativum*) was the best terminator [64].

### 3.8. Configuration of gRNA and Cas9 Cassettes

**Figure 2:**
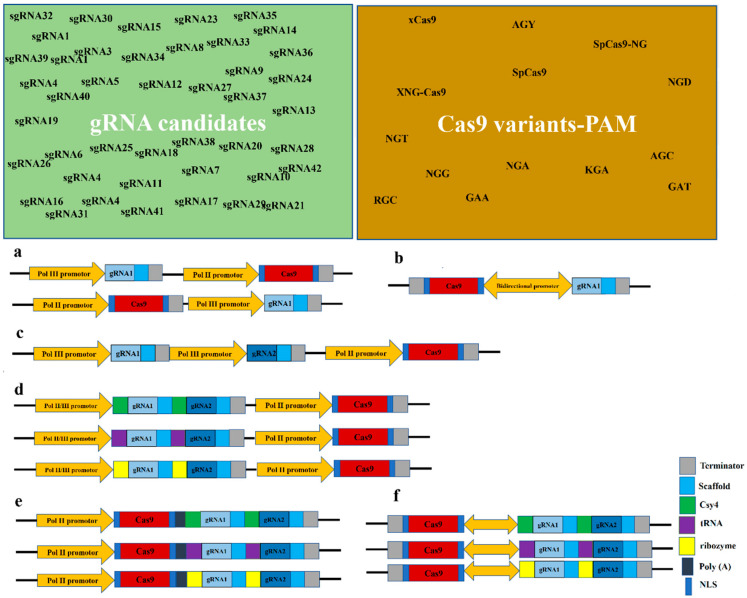
The multi-factorial nature of CRISPR/Cas9-based genome editing procedure considering configuration of gRNA and Cas9 expression cassettes along with different RNA processing strategies in multiplex gRNA experiments. (**a**) Simple TCTU configuration of gRNA and Cas9 cassettes in left and right borders of T-DNA. (**b**) Expression of gRNA and Cas9 cassettes in bidirectional promoter system. (**c**) Simple multiplex gRNA in TCTU system. (**d**) Multiplex gRNA in TCTU system with Csy4, tRNA, and ribozyme processing machines. (**e**) Multiplex gRNA in STU configuration with Csy4, tRNA, and ribozyme processing machines. (**f**) Bidirectional configuration of Cas9 and multiplex gRNA with Csy4, tRNA, and ribozyme processing machines.

Overall, there are three options to achieve co-expression of the gRNA and Cas9 cassettes: (i) a single transcriptional unit (STU), (ii) a two-component transcriptional unit (TCTU), or (iii) a bidirectional promoter system [93,94]. In the STU approach, the expression of both cassettes is jointly driven by a single Pol II promotor. In conventional TCTU, the gRNA is under the control of a pol III promoter (U3 or U6), and the Cas9 cassette is driven by a pol II promoter [94]. When inducible or tissue-specific expression is needed, an STU approach is best [95], although there can be negative impacts of such a configuration on Cas mRNA maturation and gRNA stability [94]. In a comparison of the STU and TCTU configurations in soybean, TCTU was proven to be a better option than STU [96]. Generally, the editing rate is target-dependent; therefore, finding the best configuration for optimized co-expression of the gRNA and Cas9 requires trial and error [93]. Overall, these various possible components and configurations offer a range of possibilities but also make the optimization of a CRISPR/Cas system complex and multi-factorial (Figure 2).

## 4. Examples of *Agrobacterium rhizogenes*-Mediated Delivery of CRISPR/Cas9 Reagents for Gene Functional Analysis in Soybean

Both in vitro and in planta *Agrobacterium* inoculation techniques have been applied for transient expression of CRISPR/Cas9 reagents to functional analysis of different genes in soybean hairy roots. These genes are mainly involved in nitrogen fixation/nodule development [97,98,99,100] and the interaction of soybean with pathogens [101,102]. The complete list of recently reported functional analyses is presented in Table 1. Different modifications have been used in both in vitro and in planta inoculation techniques. Some examples are provided here. The details of these examples can act as a quick reference for future experiments. 

### 4.1. Modifications in In Vitro Inoculation

Michno et al. [104] applied in vitro vacuum infiltration (constant vacuum for five minutes and held under a sealed vacuum for another 20 min) on seven-day-old cotyledon explants of soybean submerged in an *A. rhizogenes* suspension culture (OD600 = 0.2–0.3 of ARqual strain) for transient expression of CRISPR/Cas9 reagents. Infected cotyledons were co-cultivated with *Agrobacterium* on sterile filter paper at 28 °C for three days, then washed with ¼ MS liquid media supplemented with carbenicillin and transferred to ¼ MS solid media (with carbenicillin). The authors reported the emergence of hairy roots approximately fourteen days after inoculation. Therefore, the established protocol offers a rapid turn-around time in generating transgenic material, which is useful for testing various configurations of CRISPR/Cas9 components [104]. Gentle shaking (20 min at 80 rpm) of cotyledon explants in an *A. rhizogenes* suspension (OD600 = 0.2–0.8) has been reported for the delivery of different CRISPR/Cas9 constructs in soybean [103]. Acetosyringone, a phenolic compound that enhances the transfer of T-DNA to plant cells [114], can also be used during *A. rhizogenes* inoculation and/or co-cultivation steps. Jacobs et al. [103] applied 100 μM acetosyringone during *A. rhizogenes* co-cultivation (three days on filter paper) of cotyledon explants in soybean and reported 95% transformation efficiency. The same protocol was applied by Carrijo et al. [96], and an indel frequency of 73.20% was reported. In another study, different additives, including sucrose (87.64 mM) + MES (1.88 mM) + acetosyringone (0.2 mM) + L-cysteine (1.66 mM) + DTT (1 mM), were included in the *A. rhizogenes* suspension (OD600 = 0.5). The authors reported successful transformation following a 30 min *Agrobacterium* inoculation and a five-day co-cultivation period on sterile filter paper [96].

### 4.2. Modifications in in Planta Inoculation

Zheng et al. [73] dipped the sterilized blades of scissors into an *Agrobacterium* suspension (strain K599) and then cut the cotyledons of soybean cultivar Williams 82. The cut cotyledons were then transferred to sterile paper towels pre-soaked in 1/4 Gamborg’s liquid medium supplemented with 200 mg/mL Timentin. Hairy roots were ready for further analysis after 2∼3 weeks of cultivation in a growth chamber with a photoperiod of 16/8 h light/dark at 28/24 °C. A high frequency of mutation induction was reported for this protocol (80–100%).

## 5. Conclusions

**Figure 3:**
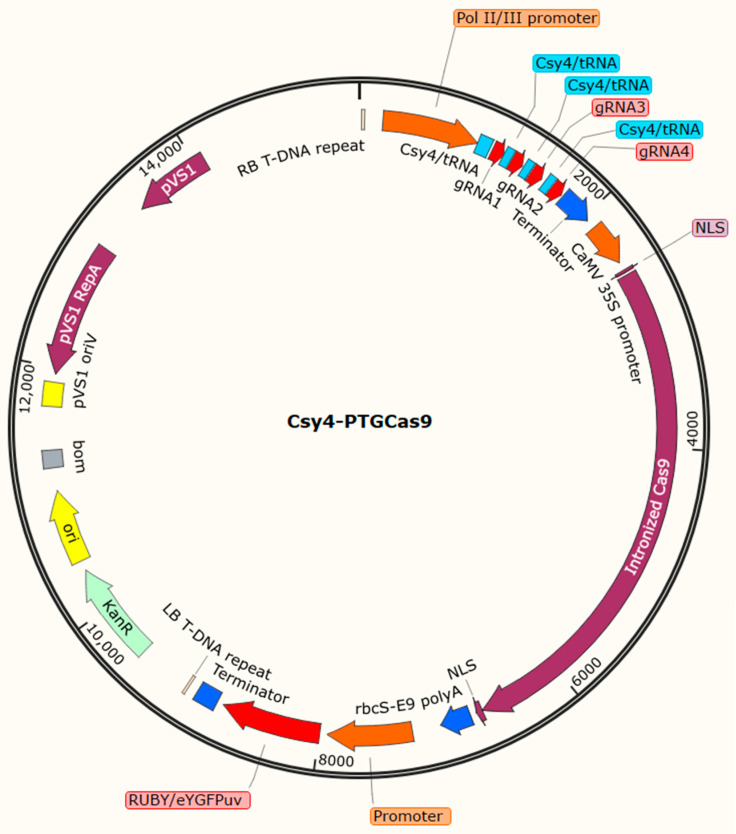
Schematic representation of an ideal vector with Csy4-gRNA/tRNA-gRNA processing systems and TCTU configuration of gRNA and Cas9 cassettes for multiplexed “in root” gene functional analysis of soybean using CRISPR/Cas9-hairy root system.

**Figure 4:**
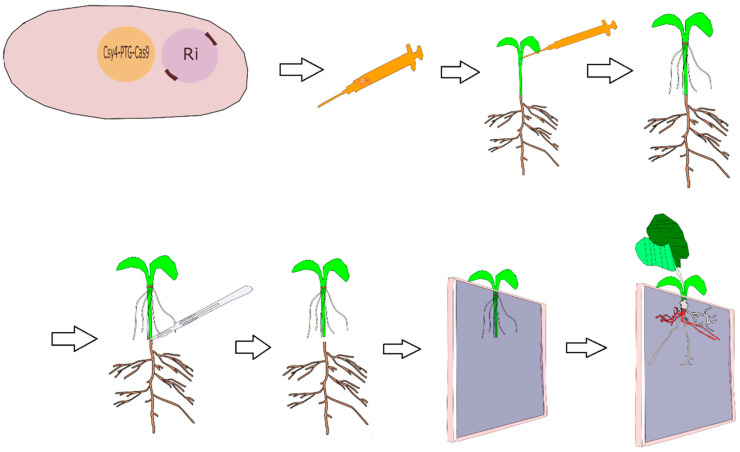
The whole procedure of *Agrobacterium rhizogenes*-mediated hairy root transformation of soybean cotyledonary node with CRISPR/Cas9 system and real-time monitoring of transformed hairy roots using *RUBY* reporter gene for efficient “in root” gene functional analysis.

An improved understanding of gene function and regulation can help usher in a new era in the improvement of crops. It is valuable to achieve a sustainable food supply in a world under the pressure of climate change and an ever-increasing population. In the genomics era, fast and reliable reverse-genetics techniques are needed to validate the large number of candidate genes identified through the large-scale analysis of genomic and transcriptomic data generated by NGS-based forward genetic techniques. Therefore, forward and reverse genetic approaches can work together in a complementary manner for the identification and validation of candidate genes and genetic components. Hairy root transformation provides a great opportunity to enable the functional validation of genetic components and candidate genes, at least in cases where the root provides an appropriate tissue for functional analysis. In planta *Agrobacterium rhizogenes*, armed with a CRISPR/Cas9 vector (multiplexed gRNAs in a Csy4/PTG manner + a soybean codon-optimized intronized Cas9 with two NLSs in both its C- and N- terminals + a *RUBY*/*eYGFPuv* reporter gene) (Figure 3), is the most rapid, precise, easy, and cost-effective method for “in-root” functional analyses in soybean (Figure 4). Nonetheless, as was reviewed above, careful design and due consideration must be given to a multitude of parameters to ensure the desired outcome and efficient and precise editing of the targeted sequence.

## Figures and Tables

**Table 1:** Examples of CRISPR/Cas9-based *A. rhizogenes*-mediated hairy root transformation for functional analysis of different genes in soybean genome.

Cultivar/Line	*Agrobacterium* *rhizogenes* strain	Targeted Gene(s)	Inoculation Technique	Explant Type	Supplementary Treatment	Massive Hairy Root Formation	Efficiency	Reference
Williams 82	NCPPB2659 (K599)	GmFEI2, GmSHR	In vitro	Cotyledonary node with hypocotyl	Acetosyringone	2 weeks	0.6–0.95%	[28]
Jack	NCPPB2659 (K599)	Glyma01g38150, Glyma11g07220	In vitro	Cotyledonary node	Acetosyringone	-	95%	[103]
Bert	ARqual	GS1, Glyma.18g041100	In vitro	Cotyledon	Vacuum infiltration	-	-	[104]
Williams 82	NCPPB2659 (K599)	Glyma06g14180, Glyma08g02290, Glyma12g37050	Ex vitro	Hypocotyls	-	-	-	[69]
Jack	NCPPB2659 (K599)	GmPDS11, GmPDS18	In vitro	Cotyledons	Acetosyringone	2 weeks	11.7–48.1%	[92]
Hill	NCPPB2659 (K599)	Glyma.01G165800, Glyma.01g165800-D	Ex vitro	Cotyledonary node	-	-	-	[105]
Williams 82	NCPPB2659 (K599)	Rfg1	Ex vitro	Cotyledonary node	-	2–3 weeks		[97]
Williams 82	NCPPB2659 (K599)	GmMYB118	Ex vitro	Cotyledonary node	-	-	50%	[106]
Williams 82	NCPPB2659 (K599)	Glyma03g36470, Glyma14g04180, Glyma06g136900	Ex vitro	-	-	-	2.8–20.6%	[107]
-	NCPPB2659 (K599)	-	In vitro	Cotyledons	Gentle shaking	3 weeks	-	[108]
Williams 82	NCPPB2659 (K599)	GmLCLa1, GmLCLa2, GmLCLb1, GmLCLb2	In vitro	Cotyledons with hypocotyls	-	-	-	[109]
Williams 82	NCPPB2659 (K599)	GmAGO7a, GmAGO7b	Ex vitro	Cotyledonary node	-	2–3 weeks	80–100%	[73]
LD10-30110	ARqua	Glyma.15G191200	In vitro	Cotyledon	Acetosyringone, cysteine, sodium thiosulfate	-	-	[110]
Jack	NCPPB2659 (K599)	GmIPK1, GmIPK2	In vitro	Cotyledonary node	Acetosyringone	-	73.20%	[96]
Williams 82, Magellan, Zhonghuang13, Maverick	NCPPB2659 (K599)	GmNSF, GmSNAP	In vitro	Cotyledon	Acetosyringone, 6-BA, GA3	20 d	69%	[34]
Fayette	NCPPB2659 (K599)	DELLA11, DELLA18	In vitro	Cotyledons	-	-	-	[101]
Williams 82	NCPPB2659 (K599)	GmROP6a/b, GmROP9a/b	Ex vitro	Hypocotyls	-	2–3 weeks	21–43%	[98]
Williams 82	NCPPB2659 (K599)	miR156a, miR156c, miR156f, miR166a, miR167a, miR172a, miR172b, miR172c, miR172d, miR2118a, miR396a, miR396c, miR396e, miR397a, miR398a, miR399d, miR408a, FEI, NARK	Ex vitro	Hypocotyls	-	2–3 weeks	-	[46]
Williams 82		NSP1a, NSP1b	Ex vitro	Hypocotyls	-	-	-	[82]
Williams 82	NCPPB2659 (K599)	GmNAC06	Ex vitro	Cotyledonary node	-	4 weeks	-	[111]
Williams 82	NCPPB2659 (K599)	Glyma.15G249000, Glyma.13G259100	In vitro	Seed	-	25 days	45.3%	[80]
Williams 82		GmUOX, GmXDH	Ex vitro	-	-	-	54%	[99]
Mustang	NCPPB2659 (K599)	GmNHX5	In vitro	Cotyledonary node	MES + acetosyringone	15 d	-	[35]
Tianlong 1	NCPPB2659 (K599)	GmpPLA-IIε, GmpPLA-IIζ	-	-	-	-	-	[112]
Williams 82	NCPPB2659 (K599)	GmNFYA-C, miR169c	In vitro	Cotyledonary node	Ammonium glufosinate	-	-	[113]
Tianlong 1, Suinong 10	NCPPB2659 (K599)	GmDRR1	Ex vitro	Cotyledonary node	-	-	-	[102]
Williams 82	NCPPB2659 (K599)	GmSPL9d, miR156	Ex vitro	Cotyledonary node	-	-	-	[100]

## Data Availability

Data sharing not applicable to this article as no datasets were generated nor analyzed during the current review study.

## Abbreviations

ABE	Adenine base editor
BE	Base editor
CBE	Cytosine base editor
CRISPR/Cas	Clustered regularly interspaced short palindromic repeats/CRISPR-associated protein
DSB	Double-strand breaks
gRNA	Guide RNA
NGS	Next-generation sequencing
NLSs	Nuclear localization signals
PAM	Protospacer adjacent motif
PTG	Polycistronic tRNA-gRNA vector
RNAi	RNA interference
sgRNA	Single guide RNA
TALENs	Transcription activator-like effector nucleases
ZFNs	Zinc finger nucleases
